# Mind and Body: Italian Validation of the Postural Awareness Scale

**DOI:** 10.3389/fpsyg.2020.00827

**Published:** 2020-05-18

**Authors:** Eleonora Topino, Alessio Gori, Holger Cramer

**Affiliations:** ^1^Department of Human Science, LUMSA University, Rome, Italy; ^2^Department of Health Sciences, University of Florence, Florence, Italy; ^3^Department of Internal and Integrative Medicine, Evang. Kliniken Essen-Mitte, Faculty of Medicine, University of Duisburg-Essen, Essen, Germany

**Keywords:** posture, awareness, mind–body integration, Italian validation, self-report questionnaire

## Abstract

Postural awareness (PA) refers to a subjective conscious awareness of body posture and falls within the framework of mind–body integration. The aim of this research was to validate and evaluate psychometric properties of the Postural Awareness Scale (PAS) in an Italian population sample (*n* = 928; 45.04% men and 54.96% women; mean age = 29.96 years, standard deviation = 11.44). The results obtained with Velicer’s Minimum Average Partial Test, Horn’s Parallel Analysis, and exploratory factor analysis showed a two-factor solution, as supported by the confirmatory factor analysis: ease/familiarity with postural awareness and need for attention regulation with postural awareness. Furthermore, the findings highlighted both a good internal consistency (α = 0.76 for the total scale and α = 0.80, α = 0.79 for the two subscales) and a satisfactory construct validity. Furthermore, multivariate analysis of variance was carried out to assess differences in PA between specific subgroup. In particular, the positive effects of physical activity and healthy body weight were confirmed, whereas no significant differences related to gender or age were found. All these findings suggest that the Italian version of the PAS is a rapid instrument with good psychometric properties, which can be useful both for research and clinical practice.

## Introduction

The postural awareness is “the subjective conscious awareness of body posture that is mainly based on proprioceptive feedback from the body periphery to the central nervous system” (Cramer et al., 2018b, paragraph 1). It is a fundamental element for controlling posture in a process of adaptation based on sensory, motor, and cognitive aspects (Balasubramaniam and Wing, 2002). The body posture, in fact, can be influenced by a certain number of conditioning factors: mechanical aspects, heredity, race, flexibility, muscular strength, vision, and habits, but it is also involved in relationships of mutual interdependence with emotional and psychological factors (Brito, 1995; Wright et al., 2000). The scientific literature confirms the close relationship between posture and psychological dimensions, as demonstrated in several studies concerning assertiveness levels and action trends (e.g., Maner et al., 2010; Huang et al., 2011; Arnette and Pettijohn, 2012; Van der Toorn et al., 2015), self-efficacy (Anderson and Galinsky, 2006), self-esteem (Stepper and Strack, 1993; Dijkstra et al., 2007; Kwon and Kim, 2015), and mood (Hackford et al., 2019). These findings fall within a framework of mind–body interaction supported by different lines of research. The field of trauma studies, for example, increasingly focuses on this reciprocal influence, describing somatically driven individuals, with strong emotions accompanied by dysregulated physical sensations: these activations derive from reminders to previous adverse experiences that were not elaborated and repeated in the body (Van Der Kolk, 2014; Fisher, 2017). Furthermore, trauma affects self-awareness, specifically the sensory one (Bluhm et al., 2009), and alters the “postural body scheme” (Gurfinkel, 1994), involved in the perception of self with respect to the external world and in the actions directed to it. All this seriously alters the psychological perception of being able to manage one’s life, which is closely linked to the possibility of experiencing control of the physical sphere (Van Der Kolk, 2008). Therefore, maintaining or increasing one’s postural awareness levels allows the management of one’s “postural body scheme,” developing more adaptive attitudes through reflection and intention (Massion, 1994). Thus, the vision of James Grotstein (1997) who spoke of the mind and the body as a “strangely coupled unity” appears pertinent. He depicts them in a single entity with two inseparable aspects, like two sides of the same coin: they are considered like two different categories dependent on the perspective of the observer (Solano, 2010). In this theoretical framework, body awareness and mindfulness are parallel to the construct of postural awareness and strongly associated with it.

Body awareness concerns attention to bodily sensations and implies access to consciousness of proprioceptive (including posture) and interoceptive aspects (Mehling et al., 2009). It allows the participation of bodily sensations in everyday life and the observation of changes and physical responses to emotions and environment. It finds a good application and positive feedback in many contexts of clinical care such as, for example, those for recovery from physical and/or psychological traumas (Herman, 1992; Bishop et al., 2004), for substance abuse (Marlatt and Ostafin, 2005), for eating disorders (Zerbe, 1995), and for personality disorders (Friis et al., 1989). On the contrary, the concept of bodily dissociation is characterized by the avoidance of inner experience (Price and Thompson, 2007); it could represent a protective strategy against painful memories, thoughts, or feelings and is a mechanism commonly used for defense against physical suffering (Bakal, 2001) and trauma (Van der Kolk, 2006).

Closely associated, mindfulness is an awareness of the present moment with total acceptance of it (Brown and Ryan, 2003). Mindfulness intertwines focused attention with meta-awareness, allowing deep insight and clarifying the nature of the elements that constitute the experience (Wallace, 2006). This presence disposition is closely connected to higher levels of physical and mental health, better postural control through the conscious management of attentional focus (Kee et al., 2012), and more likely to maintain healthy habits such as sufficient sleep (Roberts and Danoff-Burg, 2010), physical activity (Murphy et al., 2012), and healthy eating (Gilbert and Waltz, 2010).

In this frame of mind–body relationship, postural awareness has still been little investigated, even though it is related to other important constructs explored above. Some studies support its effectiveness in chronic pain situations: in this field, considering the impact of this condition on people’s lives and the psychological difficulties that ensue, new treatment models have been developed based on the association of physical experiences to states of greater awareness and mindfulness (Mattsson, 1998; Rosberg, 2000). Specifically, postural awareness training has proved particularly effective for chronic low back pain conditions (Moseley et al., 2004; Ahmed et al., 2016). These favor the control of one’s physical disposition and the maintenance of healthy postural patterns in everyday life, important elements to avoid chronicization and further deterioration (Cramer et al., 2018b); a faulty posture, in fact, increases stress on muscles, tendons, ligaments, and bones (Yamak et al., 2018).

### The Postural Awareness Scale: A Measure of Body Posture Awareness

The Postural Awareness Scale (PAS) is a German self-report measure designed by Cramer et al. (2018b), which allowed them to grasp the increases of this variable on subjects with chronic pain following the implementation of a multimedia mind–body training program. In particular, they found that improvements in body posture awareness were longitudinally associated with reduced pain in patients with spinal/shoulder pain, in line with other research on this topic (e.g., Lauche et al., 2017). The scale consists of 12 items, grouped into two factors (explaining the 58.8% of the variance in the original study); the first one is “ease/familiarity with postural awareness,” which refers to an effortless awareness and connectedness; the second factor is “need for attention regulation with postural awareness” and indicates a forced awareness. The original scale and both its factors demonstrated satisfactory internal consistency and good validity converging with other measures related to body awareness and mindfulness. Specifically, the subscale *ease/familiarity with postural awareness* showed important associations with the measures related to the connection with one’s body (Cramer et al., 2018b), significantly correlating with the scores of the Body Awareness Questionnaire (BAQ; Shields et al., 1989), of the *trust in bodily sensations* subscale [Body Responsiveness Questionnaire (BRS); Daubenmier, 2005; Cramer et al., 2018a], and of the Conscious Presence and Self-Control scale (Büssing et al., 2013). The *need for attention regulation with postural awareness* subscale, on the other hand, did not significantly correlate with the BAQ, but showed a relevant association with the BRS Perceived Connection between Mental and Physical Process subscale, reflecting the need to strive for achieving or maintaining a link between cognitive process and bodily needs. To conclude, both factors were also significantly correlated to the subscales of the Dresden Body Image Inventory (Pöhlmann, 2014), indicating the association between high levels of posturalawareness and a more positive attitude toward one’s body and appearance.

### Rationale for the Study

Further studies on postural awareness would add useful contributions to the mind–body integration perspective, with possible positive repercussions in the field of psychological and psychotherapeutic intervention. The scientific literature shows the efficacy of some interventions for the improvement of posture aspects such as balance (Wayne et al., 2004; Kee et al., 2012), coordination (Jay et al., 2013), control (Pluchino et al., 2012), and awareness (Roll et al., 2002). However, up to now, particularly complex (Barrus, 1996; Aminian and Najafi, 2004) and/or hardly usable outside the laboratory setting (Lanningham-Foster et al., 2005; Wong et al., 2007) tools have been used to measure these outcomes. With the exception of PAS, no self-report tools have been found to allow a more agile assessment of subjective postural awareness (Cramer et al., 2018b). The simplicity of this self-administered scale would enable a measurement of postural awareness in the absence of technical devices and within a psychological setting.

The aim of the present research is the validation (and evaluation of psychometric properties) of the Italian PAS, originally created in German by Cramer et al. (2018b), to allow its use in research and clinical practice. In light of the excellent psychometric characteristics of the original instrument, we hypothesize to obtain an Italian version with a good internal coherence and a similar and equally good factor structure.

## Materials and Methods

### Participants

The study involved 928 individuals (45.04% men and 54.96% women) with an age ranging from 18 to 77 years (mean = 29.96, standard deviation = 11.44). The sample included participants from Northern (37.50%), Central (32.54%), and Southern (29.96%) Italy. Most individuals were unmarried (71.55% single). Of the 928 participants, 456 (49.14%) were students, and 255 (27.48%) were employed; 44.61% of them held a secondary school diploma, 27.37% a bachelor’s, and 19.83% a master’s degree; 48.28% of the sample was Catholic Christian, and 45.37% was atheist. Three hundred seventy-four participants (40.30%) did not practice any type of sports, whereas 260 (28.02%) trained in the gym (Table 1).

**TABLE 1:** Demographics variables of the sample (*N* = 928).

Age		
	Mean = 29.96, Standard deviation = 11.44	

	*n*	%
**Sex**		
Male	418	45.04
Female	510	54.96
**Provenance**		
Northern Italy	348	37.50
Central Italy	302	32.54
Southern Italy	278	29.96
**Marital status**		
Single	664	71.55
Married	111	11.96
Separated	34	3.66
Divorced	22	2.37
Widowed	11	1.19
Cohabitant	86	9.27
**Professional condition**		
Unemployed	64	6.90
Student	456	49.14
Housewife	12	1.23
Freelance	123	13.25
Employee	255	27.48
Retired	10	1.08
Other	8	0.86
**Study degree**		
Middle school diploma	52	5.60
High school diploma	414	44.61
University degree	254	27.37
Master’s degree	184	19.83
Postlaurea specialization	24	2.59
**Religion**		
Catholic Christian	448	48.28
Muslim	2	0.22
Buddhist	11	1.19
Atheist	421	45.37
Jehovah’s Witness	3	0.32
Agnostic	30	3.23
Other	13	1.40
**Sport**		
Gym	260	28.02
Water sports	46	4.96
Football/soccer	34	3.66
Cycling and running	31	3.34
Walk and trekking	27	2.91
Bodyweight exercises, free exercises, yoga, fitness	35	3.77
Dance and skating	23	2.48
Volley	20	2.16
Basket and rugby	16	1.72
Martial arts and combat sports	33	3.56
Other	29	3.13
No sport	374	40.30

### Procedures

Items of the original version of the PAS have been translated into Italian by a native German speaker living in Italy. Then, the Italian version was back-translated by a bilingual Italian German teacher, and the outcome was submitted to the author of the original measure, with the help of which the remaining inaccuracies were corrected. The researchers compared the translated version with the original text until a consensus on cross-language equivalence was reached. The participants were recruited on the internet with an anonymous link spread through a snowball-like procedure, and the presence of psychological or orthopedic issues was adopted as criteria for exclusion from the sample. All the subjects were informed about the aim of the research and gave written informed consent in accordance with the Declaration of Helsinki. The self-report measures together with a demographic questionnaire (i.e., age, sex, weight, height) were administered to participants, who did not take any compensation for their involvement in the study. The subjects were guaranteed privacy and anonymity.

### Measures

#### Postural Awareness Scale

The PAS is a brief self-report measure designed to assess awareness of body posture (Cramer et al., 2018b), and it consists of 12 items scored on a 7-point scale anchored by 1 (not at all true for me) and 7 (very true for me). Results supported the internal consistency of the original German PAS, with a Cronbach α of 0.80 for the total scale and 0.81 and 0.77 for the two subscales (*ease/familiarity with postural awareness* e *need for attention regulation with postural awareness*, respectively). The scale scores range from 12 to 84, with higher scores being indicative of greater postural awareness. The scores were computed by adding up the answers to all the items, after reversing the values of items 1, 2, 3, 4, 5, and 12. In this study, an Italian version obtained by a back-translation process was used.

#### Body Image Concern Inventory

The Body Image Concern Inventory (BICI) is a self-report measure for assessing experiences related to dysmorphic concern (Littleton et al., 2005). In this study, the Italian version of the BICI (I-BICI; Luca et al., 2011) was used. It consists of 19 items divided into two subscales: dysmorphic symptoms and symptom interference. Response categories ranged from 1 (never) to 5 (always), and the scale scores range from 19 to 95. The aspects investigated were dissatisfaction and concern about appearance, checking and camouflaging behavior, reassurance seeking, social concerns, and avoidance related to appearance. In this sample, the I-BICI possesses good internal consistency, with a Cronbach α of 0.92 and 0.76 for the two subscales and α = 0.93 for the total scale.

#### Rosenberg Self-Esteem Scale

The Rosenberg Self-Esteem Scale (RSES) is a 10-item self-report questionnaire designed for assessing global self-esteem with items answered on a 4-point scale from *strongly agree* to *strongly disagree* (Rosenberg, 1965). The scale scores range from 0 to 30, in which scores between 15 and 25 are within normal range, whereas scores less than 15 suggest low self-esteem. In this study, the Italian version of the RSES (Prezza et al., 1997), showing good internal consistency (α = 0.90), was used.

#### General Self-Efficacy Scale

The General Self-Efficacy Scale (GSE) is a self-report measure of self-beliefs to cope with a variety of difficult demands in life (Schwarzer and Jerusalem, 1995). It consists of 10 items scored on a 4-point scale anchored by 1 (not at all true for me) and 4 (very true for me). The scale scores range from 10 to 40, with higher scores being indicative of a sense of personal competence in stressful situations. In this sample, the Italian versions of the GSE (Sibilia et al., 1995) showed a high internal consistency (α = 0.90).

#### Body Awareness Questionnaire

The BAQ is an 18-item self-report questionnaire designed to assess the sensitivity to normal and non-emotional body processes (Shields et al., 1989). Each item on the measure is rated on a 7-point scale ranging from 1 (not at all true for me) to 7 (very true for me). In this study, the Italian translation of BAQ (Shields et al., 1989; for the Italian version Cardinali, unpublished manuscript) possesses good internal consistency with a Cronbach α of 0.88.

#### West Haven–Yale Multidimensional Pain Inventory – Short Version

The West Haven–Yale Multidimensional Pain Inventory (WHYMPI-S) is a self-report measure designed to examine the impact of chronic pain on patients’ lives, quality of social support, and general activities (Kerns et al., 1985). In the present study, a short version of this measure was used: five items (2, 8, 9, 12, 19) of the 52 taken from the Italian version (Ferrari et al., 2000), showing a good internal consistency (α = 0.87), were readapted. The selected items evaluated interference in daily life, changes in the ability to participate in recreational and social activities, in the level of satisfaction deriving from involvement in family activities, in the level of suffering, and in friendship. Responses were on a 5-point Likert scale, and higher scores indicated higher levels of suffering and impact of chronic pain.

#### 20-Item Toronto Alexithymia Scale

The 20-item Toronto Alexithymia Scale (TAS-20) is a well-known 20-item questionnaire, scored on a 1- to 5-point Likert scale, which assesses the level of alexithymia (Bagby et al., 1994). The scale measures three main dimensions: (1) difficulty in identifying feelings and distinguishing between feelings and bodily sensations in emotional activation, (2) difficulty in the verbal expression of emotions, and (3) externally oriented thinking. In this sample, the Italian version of the TAS-20 (Bressi et al., 1996), showing a good internal consistency with a Cronbach α of 0.86 for the total score (α = 0.84, 0.79, 0.65 for the subscales), was used.

#### Beck Depression Inventory II

The Beck Depression Inventory II (BDI-II) is a 21-item self-report multiple-choice inventory designed to assess the intensity of depression (Beck et al., 1996). Response categories range from 1 to 3, and the scale scores range from 0 to 63. It is composed of two subscales: a cognitive–affective and a somatic–performance subscale. In this study, the Italian translation of BDI-II (Ghisi et al., 2006) possesses high internal consistency with a Cronbach α of 0.91 for the total score (α = 0.84 and 0.88 for the subscales).

#### Mindfulness Attention Awareness Scale

The Mindfulness Attention Awareness Scale (MAAS) is a self-report measure designed to assess present attention and awareness (Brown and Ryan, 2003). In this study, the Italian version of the MAAS (Veneziani and Voci, 2015) was used. It includes 15 items to be rated on a 7-point Likert scale from 1 (almost always) to 7 (almost never), with higher scores being indicative of greater mindfulness. In this sample, the Italian version possesses a good internal consistency (α = 0.87).

#### Modified Somatic Perception Questionnaire

The Modified Somatic Perception Questionnaire (MSPQ) is a self-report measure of somatic and autonomic perceptions (Main, 1983). In this study, the Italian translation of MSPQ (Conti, 1999) was used. It consists of 22 items scored on a 0- to 4-point Likert scale, 13 of which are used for the final score (the others have a masking function). In the present sample, the Italian version possesses a good internal consistency (α = 0.85).

### Data Analysis

All the statistical analyses were performed using the software SPSS 25.0 for Windows (IBM Corp, 2017, Armonk, NY, United States) and MPlus Version 8.1 (Muthén and Muthén, 1998–2017). Descriptive statistics were examined. To test the factor structure of the Italian PAS, the sample was randomly split. On the first subsample, Velicer’s Minimum Average Partial Test (MAP), Horn’s Parallel Analysis (HPA), and an exploratory factor analysis (EFA) with principal axis factoring extraction method (Promax rotation) were performed. Then, the factor structure was verified with a confirmatory factor analysis (CFA) on the second subsample, using the following fit indices: (1) the model χ^2^, which indicates a good model fit when *p* > 0.05 (Hooper et al., 2008); (2) the goodness-of-fit statistic (GFI), with recommended values ≥ 0.95 (Hooper et al., 2008); (3) the non-normed fit index (NNFI) with recommended values ≥ 0.95 (Hu and Bentler, 1999); (4) the comparative fit index (CFI), for which the recommended values are ≥ 0.95, although values between 0.90 and 0.95 indicate reasonable fit (Kline, 2005); (5) the root mean square error of approximation (RMSEA), with recommended values ≤0.05, although values up to 0.08 represent reasonable errors of approximation (Marsh et al., 2004); (6) the standardized root mean square residual, with recommended values ≤0.08 (Hooper et al., 2008; Hu and Bentler, 1999). After that, the reliability of the scale was calculated both with the Cronbach α coefficient and item-total correlation indices. In order to assess some aspects of construct validity, Pearson correlation was calculated between PAS, I-BICI, RSES, GSE, BAQ, WHYMPI-S, TAS-20, BDI-II, MAAS, and MSPQ. The choice of these measures was driven by the observation that there are no other self-report questionnaires for the assessment of postural awareness: measures evaluating aspects of awareness and somatic perceptions were therefore included. Moreover, as for large samples even low correlations could be significant, greater precision was searched in the evaluation of the discriminating validity of the two subscales of the PAS, by implementing a correlation coefficients comparison according to Meng et al. (1992). Finally, to assess the differences between specific subgroups, the multivariate analysis of variance (MANOVA) was carried out, by simultaneously entering all the background variables [gender, age, practice of sport, body mass index (BMI)] as fixed factors in a multivariate general linear model. Separate follow-up ANOVAs were conducted for the dependent variables when it was necessary, and *post hoc* analyses using Scheffé test were performed to support the interpretation of the differences between averages where needed.

## Results

### Descriptive Statistics

The descriptive statistics of the sample were reported in Table 1. The mean values of the PAS items ranged from 2.69 to 5.61 (Table 2).

**TABLE 2:** Descriptive statistics and item-total correlations of each of the Italian PAS items.

					Standard			Item-total
Item	*N*	Minimum	Maximum	Mean	deviation	Skewness	Kurtosis	correlation
1^a^	928	1.00	7.00	4.44	1.76	–0.13	–0.91	0.49
2^a^	928	1.00	7.00	3.88	1.95	0.10	–1.23	0.42
3^a^	928	1.00	7.00	3.27	2.05	0.56	–0.98	0.36
4^a^	928	1.00	7.00	2.69	1.69	1.01	0.25	0.34
5^a^	928	1.00	7.00	4.30	2.08	–0.15	–1.33	0.50
6	928	1.00	7.00	3.71	1.84	0.12	–1.08	0.38
7	928	1.00	7.00	4.61	1.93	–0.46	–0.90	0.19
8	928	1.00	7.00	3.47	1.75	0.32	–0.91	0.57
9	928	1.00	7.00	4.54	1.72	–0.35	–0.79	0.41
10	928	1.00	7.00	3.08	1.69	0.53	–0.65	0.56
11	928	1.00	7.00	3.58	1.88	0.21	–1.05	0.29
12^a^	928	1.00	7.00	3.90	1.87	0.17	–1.01	0.26
Valid *N* (listwise)	928							

*^*a*^Reversed scoring.*

### Factor Structure of the Italian PAS

First, in accordance with O’connor (2000), the MAP and HPA were carried out (Table 3). Both the original MAP (Velicer, 1976) and the revised MAP (Velicer et al., 2000) suggested the retention of two factors, as well as the HPA (Horn, 1965).

**TABLE 3:** MAP test and parallel analysis results for the number of components.

	Average partial correlations				Random data eigenvalues	
*N*	Squared	Power 4	*N*	Eigenvalues	Means	95% Percentile
0	0.08	0.01	1	3.34	1.27	1.32
1	0.07	0.01	**2**	**2.78**	**1.20**	**1.25**
**2**	**0.03**	**0.00**	3	0.96	1.14	1.20
3	0.04	0.00	4	0.83	1.10	1.13
4	0.05	0.01	5	0.66	1.05	1.08
5	0.07	0.01	6	0.64	1.02	1.04
6	0.10	0.03	7	0.61	0.98	1.01
7	0.14	0.06	8	0.56	0.93	0.97
8	0.22	0.11	9	0.54	0.89	0.93
9	0.31	0.20	10	0.42	0.86	0.89
10	0.60	0.47	11	0.39	0.81	0.84
11	1	1	12	0.28	0.76	0.81

*Bold values show the number of components, according to the tests.*

**FIGURE 1:**
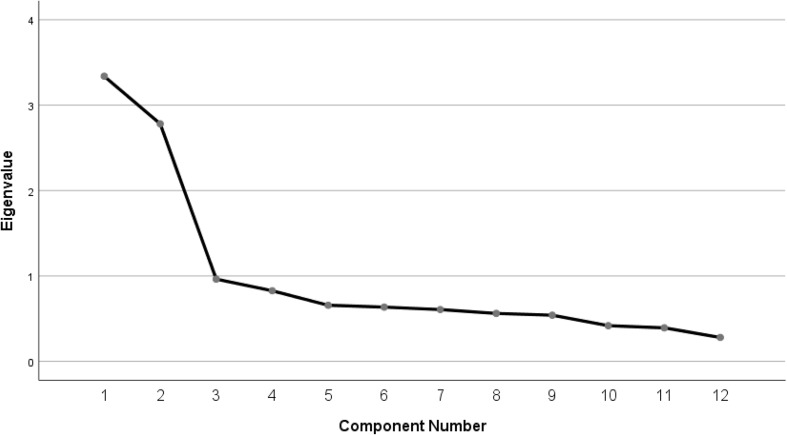
Scree plot.

Furthermore, an EFA with principal axis factoring extraction method (Promax rotation) yielded two interpretable factors, which explained 51.00% of the total variance (Table 4 and Figure 1). The first factor (ease/familiarity with postural awareness) was made up of six items related to high postural awareness without effort and accounted for 27.82% of the total variance. The second factor (need for attention regulation with postural awareness) consisted of six items related to high efforts required to be aware of their own body posture; it accounted for 23.18% of the total variance.

**TABLE 4:** Factor structure of the Italian PAS.

Item	F1	F2
1. Ich muss mich sehr konzentrieren, um meine Körperhaltung wahrzunehmen.^*b*^	0.24	**0.63**
(Needs to concentrate for being aware of posture)		
*Devo concentrarmi molto per percepire la mia postura*^*a,c*^		
2. Wenn ich eine ungünstige Körperhaltung einnehme, bemerke ich dies oft erst, wenn ich Schmerzen bekomme.^*b*^	0.07	**0.56**
(Awareness of bad posture only by pain)		
*Spesso, mi accorgo di assumere posture scorrette solo quando provo dolore* ^*a,c*^		
3. Im Sitzen sacke ich oft unbewusst in mich zusammen.^*b*^	0.02	**0.62**
(Slumps down when sitting)		
*Quando sono seduto/a, spesso mi “accascio” inconsapevolmente*^*a,c*^		
4. Wenn ich mich auf eine Tätigkeit konzentriere, nehme ich oft unbewusst eine bestimmte Körperhaltung ein.^*b*^	−0.09	**0.65**
(Unaware of posture when focused)		
*Mi capita spesso di assumere inconsapevolmente una determinata postura quando sono concentrato/a su un’attività*^*a,c*^		
5. Es fällt mir schwer, bewusst eine bestimmte Körperhaltung einzunehmen.^*b*^	0.25	**0.66**
(Difficulties to consciously adopt a posture)		
*Ho difficoltà ad adottare consapevolmente una certa postura^*a,c*^*		
6. Während der Arbeit überprüfe ich immer wieder meine Körperhaltung.*^*b*^*	**0.60**	0.08
(Often checks posture when working)		
*Controllo spesso la mia postura mentre lavoro*^*c*^		
7. Über meine Körperhaltung kann ich beeinflussen, wie ich auf andere Menschen wirke.^*b*^	**0.53**	−0.23
(Influences her/his own appeal by posture)		
*Attraverso la mia postura sono in grado di influenzare l’impressione che do alle altre persone*^*c*^		
8. Mir ist im Alltag immer bewusst, wie ich im Moment sitze oder stehe.^*b*^	**0.72**	0.27
(Always aware of sitting or standing posture)		
*Nella vita di tutti i giorni sono sempre consapevole di com’è la mia postura quando sono seduto/a o in piedi*^*c*^		
9. Ich rufe mir oft aktiv ins Bewusstsein, wie ich im Moment sitze oder stehe.^*b*^	**0.73**	0.04
(Often makes her/himself aware of her/his posture)		
*Spesso cerco di essere consapevole della mia postura da seduto/a o in piedi*^*c*^		
10. Selbst bei konzentrierten Arbeiten bin ich mir meiner Körperhaltung stets bewusst.^*b*^	**0.68**	0.29
(Aware of posture even when focused)		
*Sono sempre consapevole della mia postura anche quando sto svolgendo attività che richiedono concentrazione*^*c*^		
11. Über meine Körperhaltung kann ich bewusst steuern, wie es mir geht.^*b*^	**0.59**	−0.04
(Regulates how she/he feels through posture)		
*Riesco a influenzare consapevolmente come mi sento attraverso la mia postura*^*c*^		
12. Ob eine Körperhaltung mir gut tut oder nicht merke ich meist erst, wenn ich mich darauf konzentriere.^*b*^	−0.03	**0.49**
(Needs to concentrate to feel whether a posture benefits her/him or not)		
*Il più delle volte, noto se una postura va bene o meno per me solo se mi concentro su di essa*^*a,c*^		
**Factor correlation matrix**		
Factor 1	1	
Factor 2	0.14	1

*Factor 1, ease/familiarity with postural awareness (α = 0.80); Factor 2, need for attention regulation with postural awareness (α = 0.79). ^*a*^Reverse item. ^*b*^Original version of the PAS. ^*c*^Italian version of the PAS. Bold values indicate strong factor loadings.*

Concerning the CFA, although the χ^2^ was significant with χ^2^(36, *n* = 463) = 134.877, *p* < 0.001, the other indices showed satisfactory values and supported the two-factor solution of the Italian PAS: GFI = 0.954, NNFI = 0.921, CFI = 0.940, RMSEA = 0.077, SRMR = 0.066.

### Reliability of the Scale

A Cronbach α coefficient (α = 0.76 for the total scale and α = 0.80, 0.79 for the two subscales) suggested satisfactory reliability. Item-total correlations (Table 2) showed values ranging from 0.19 (Item 7) to 0.57 (Item 8).

### Construct Validity

Intercorrelations between PAS subscale scores were *r* = 0.11, *p* < 0.01, and they significantly and positively correlated with the PAS total score (F1, *r* = 0.73, *p* < 0.01; F2, *r* = 0.76, *p* < 0.01).

The Italian PAS showed significant correlations with most measures used to assess construct validity (Table 5). More specifically, correlations of particular importance for the convergent validity were those shown with BAQ (*r* = 0.23, *p* < 0.01, for the total PAS scale; and *r* = 0.32, *p* < 0.01, for the first PAS subscale, but there was no significant correlation with the second PAS factor), RSES (*r* = 0.19, *p* < 0.01; *r* = 0.07, *p* < 0.05; *r* = 0.22, *p* < 0.01 for total PAS score, the first and the second PAS subscales, respectively), GSE (*r* = 0.25, *p* < 0.01; *r* = 0.24, *p* < 0.01; *r* = 0.14, *p* < 0.01 for total PAS score, the first and the second PAS subscales, respectively), and MAAS (*r* = 0.19, *p* < 0.01; *r* = 0.13, *p* < 0.01; *r* = 0.15, *p* < 0.01 for total PAS score, the first and the second PAS subscales, respectively). Regarding discriminant validity, specific relevance has been given to I-BICI and TAS-20 measurements. The PAS total score and its second subscale were significantly and negatively correlated with the I-BICI total scale (*r* = −0.28, *p* < 0.01; *r* = −0.37, *p* < 0.01, respectively), the first I-BICI subscale (*r* = −0.28, *p* < 0.01; *r* = − 0.37, *p* < 0.01, respectively), and the second I-BICI subscale (*r* = −0.20, *p* < 0.01; *r* = − 0.28, *p* < 0.01, respectively). Furthermore, the PAS total score and its subscales were significantly and negatively correlated with the TAS-20 total scale (*r* = −0.25, *p* < 0.01; *r* = − 0.09, *p* < 0.01; *r* = −0.28, *p* < 0.01, respectively), the first TAS-20 subscale (*r* = −0.22, *p* < 0.01; *r* = −0.31, *p* < 0.01, only total PAS and the second PAS factor, respectively), the second TAS-20 subscale (*r* = −0.18, *p* < 0.01; *r* = −0.21, *p* < 0.01, only total PAS and the second PAS factor, respectively), and the third TAS-20 subscale (*r* = −0.18, *p* < 0.01; *r* = − 0.14, *p* < 0.01; *r* = −0.13, *p* < 0.01, respectively).

**TABLE 5:** Correlations of the measures used to assess construct validity.

	1	1a	1b	2	2a	2b	3	4	5	6	7	7a	7b	7c	8	8a	8b	9	10
																			
1) PAS	1	0.73** [0.69, 0.78]	0.76** [0.72, 0.80]	−0.28** [−0.34, −0.21]	−0.28** [−0.34, −0.21]	−0.20** [−0.27, −0.14]	0.19** [0.13, 0.26]	0.25** [0.19, 0.32]	0.23** [0.16, 0.29]	−0.14** [−0.20, −0.07]	−0.25** [−0.31, −0.19]	−0.22** [−0.29, −0.16]	−0.18** [−0.24, −0.12]	−0.18** [−0.24, −0.11]	−0.23** [−0.29, −0.17]	−0.24** [−0.30, −0.17]	−0.18** [−0.25, −0.12]	0.19** [0.12, 0.25]	−0.15** [−0.21, −0.09]
1a) PAS (F1)		1	0.11** [0.05, 0.18]	−0.04 [−0.10, 0.03]	−0.04 [−0.11, 0.02]	−0.02 [−0.09, 0.04]	0.07* [0.01, 0.13]	0.24** [0.18, 0.30]	0.32** [0.26, 0.38]	−0.01 [−0.08, 0.05]	−0.09** [−0.15, −0.02]	−02 [−0.09, 0.04]	−0.06 [−0.12, 0.01]	−0.14** [−0.20, −0.07]	−0.08* [−0.14, −0.01]	−0.07* [−0.14, −0.01]	−0.06 [−0.12, 0.01]	0.13** [0.06, 0.19]	−0.01 [−0.08, 0.05]
1b) PAS (F2)			1	−0.37** [−0.43, −0.31]	−0.37** [−0.43, −0.31]	−0.28** [−0.34, −0.21]	0.22** [0.15, 0.28]	0.14** [0.08, 0.20]	0.02 [−0.04, 0.09]	−0.19** [−0.25, −0.13]	−0.28** [−0.34, −0.22]	−31** [−0.37, −0.25]	−0.21** [−0.27, −0.15]	−0.13** [−0.19, −0.06]	−0.27** [−0.33, −0.20]	−0.27** [−0.34, −0.21]	−0.21** [−0.28, −0.15]	0.15** [0.09, 0.21]	−0.21** [−0.27, −0.15]
2) BICI				1	0.98** [0.98, 1.00]	0.80** [0.77, 0.84]	−0.44** [−0.50, −0.38]	−0.33** [−0.39, −0.27]	0.00 [−0.06, 0.06]	0.23** [0.17, 0.29]	0.31** [0.25, 0.37]	0.39** [0.33, 0.45]	0.26** [0.19, 0.31]	0.04 [−0.02, 0.11]	0.49** [0.43, 0.55]	0.45** [0.39, 0.50]	0.46** [0.40, 0.51]	−0.18** [−0.24, −0.11]	0.34** [0.28, 0.40]
2a) BICI (F1)					1	0.69** [0.64, 0.74]	−44** [−0.50, −0.83]	−0.33** [−0.39, −0.27]	−0.00 [−0.07, 0.06]	0.21** [0.15, 0.28]	0.30** [0.24, 0.36]	0.39** [0.33, 0.45]	0.26** [0.19, 0.32]	0.03 [−0.04, 0.09]	0.48** [0.42, 0.53]	0.44** [0.38, 0.49]	0.44** [0.38, 0.50]	−0.17** [−0.24, −0.11]	0.33** [0.27, 0.39]
2b) BICI (F2)						1	−0.34** [−0.40, −0.28]	−0.23** [−0.30, −0.17]	0.01 [−0.06, 0.07]	0.23** [0.17, 0.30]	0.25** [0.19, 0.31]	0.31** [0.24, 0.37]	0.18** [0.12, 0.25]	**0.08^∗^** [0.01, 0.14]	0.42** [0.36, 0.48]	0.37** [0.31, 0.43]	0.41** [0.35, 0.47]	−0.15** [−0.21, −0.08]	0.29** [0.23, 0.35]
3) RSES							1	0.47** [0.42, 0.53]	0.16** [0.10, 0.23]	−0.13** [−0.20, −0.07]	−0.40** [−0.46, −. 34]	−0.42** [−0.47, −0.36]	−0.32** [−0.39, −0.26]	−0.17** [−0.23, −0.11]	−0.54** [−0.60, −0.49]	−0.42** [−0.48, −0.36]	−0.59** [−0.64, −0.54]	0.22** [0.16, 0.29]	−0.23** [−0.29, −0.17]
4) GSE								1	0.26** [0.19, 0.32]	−0.14** [−0.20, −0.08]	−0.37** [−0.43, −0.31]	−0.33** [−0.39, −0.27]	−0.27** [−0.33, −0.21]	−0.26** [−0.32, −0.19]	−0.44** [−0.50, −0.38]	−0.34** [−0.40, −0.28]	−0.47** [−0.53, −0.41]	0.20** [0.13, 0.26]	−0.16** [−0.22, −0.10]
5) BAQ									1	**0.08^∗^** [0.01, 0.14]	−0.16** [−0.22, −0.09]	−0.06 [−0.13, 0.00]	−0.11** [−0.17, −0.04]	−0.22** [−0.28, −0.16]	−**0.08^∗^** [−0.14, −0.01]	−0.04 [−0.10, 0.03]	−0.11** [−0.17, −0.04]	0.11** [0.04, 0.17]	0.04 [−0.02, 0.11]
6) WHYMPI-S										1	0.20** [0.14, 0.27]	0.27** [0.21, 0.33]	0.13** [0.07, 0.19]	0.05 [−0.01, 0.12]	0.30** [0.24, 0.37]	0.34** [0.27, 0.40]	0.23** [0.17, 0.29]	−0.10** [−0.16, −0.03]	0.37** [0.31, 0.43]
7) TAS20											1	0.83** [0.80, 0.87]	0.83** [0.79, 0.87]	0.69** [0.65, 0.74]	0.49** [0.43, 0.55]	0.44** [0.39, 0.50]	0.46** [0.40, 0.52]	−0.29** [−0.35, −0.23]	0.30** [0.24, 0.36]
7a) TAS20 (F1)												1	0.59** [0.54, 0.64]	0.30** [0.24, 0.36]	0.59** [0.43, 0.64]	0.55** [0.50, 0.61]	0.52** [0.47, 0.58]	−0.27** [−0.34, −0.21]	0.40** [0.34, 0.46]
7b) TAS20 (F2)													1	0.40** [0.34, 0.46]	0.37** [0.31, 0.43]	0.31** [0.25, 0.38]	0.36** [0.30, 0.42]	−0.20** [−0.16, −0.13]	0.20** [0.14, 0.26]
7c) TAS20 (F3)														1	0.16** [0.10, 0.22]	0.13** [0.06, 0.19]	0.17** [0.10, 0.23]	−0.20** [−0.26, −0.14]	**0.07^∗^** [0.05, 0.13]
8) BDI-II															1	0.93** [0.90, 0.95]	0.92** [0.89, 0.94]	−0.28** [−0.34, −0.22]	0.49** [0.44, 0.55]
8a) BDI-II (F1)																1	0.70** [0.66, 0.75]	−0.27** [−0.34, −0.21]	0.55** [0.49, 0.60]
8b) BDI-II (F2)																	1	−0.24** [−0.30, −0.18]	0.35** [0.29, 0.41]
9) MAAS																		1	−0.18** [−0.24, −0.12]
10) MSPQ																			1
																			

**Correlation is significant at the 0.01 level (2-tailed). *Correlation is significant at the 0.05 level (2-tailed). PAS, Postural Awareness Scale; PAS (F1), Factor 1 “need for attention regulation with postural awareness”; PAS (F2), Factor 2, “ease/familiarity with postural awareness”; BICI, Body Image Concern Inventory; BICI (F1), Factor 1 “dysmorphic symptoms”; BICI (F2), Factor 2 “symptom interference”; RSES, Rosenberg Self-Esteem Scale; GSE, General Self-Esteem; BAQ, Body Awareness Questionnaire; WHYMPI-S, West Haven–Yale Multidimensional Pain Inventory – Short Version; TAS20, 20-item Toronto Alexithymia Scale; TAS20 (F1), Factor 1 “difficulty identifying feelings and distinguishing between feelings and bodily sensations in emotional activation”; TAS20 (F2), Factor 2 “difficulty in the verbal expression of emotions”; TAS20 (F3), Factor 3 “externally oriented thinking”; BDI-II, Beck Depression Inventory II; BDI-II (F1): Factor 1 “cognitive–affective subscale”; BDI-II (F2): Factor 2 “somatic–performance subscale”; MAAS, Mindfulness Attention Awareness Scale; MSPQ, Modified Somatic Perception Questionnaire. Bold values indicate significant correlations.

Then, a correlation coefficients comparison (Meng et al., 1992) was used to assess the discriminant validity of the PAS subscales (Table 6). The analysis showed that the subscales correlations with total PAS (*z* = −1.54, *p* = 0.124), the third factor of the TAS20 (*z* = −0.23, *p* = 0.817), and MAAS (*z* = −0.46, *p* = 0.644) were not significantly different.

**TABLE 6:** Comparison of correlation coefficients between PAS subscales and the other variables.

		95% Confidence interval				
	*r* Diff.	Lower limit	Upper limit	*z*	*p*	Effect size
1) PAS	–0.03	–0.15	0.02	–1.54	0.124	0.05
2) BICI	0.33	0.26	0.41	7.79	<0.001	0.26
2a) BICI (F1)	0.33	0.26	0.41	7.79	<0.001	0.26
2b) BICI (F2)	0.26	0.18	0.34	6.04	<0.001	0.20
3) RSES	–0.15	–0.24	–0.07	–3.47	<0.001	0.11
4) GSE	0.10	–0.02	0.19	2.34	0.019	0.08
5) BAQ	0.30	0.22	0.38	7.00	<0.001	0.23
6) WHYMPI-S	0.18	0.10	0.26	4.14	<0.001	0.14
7) TAS	0.19	0.11	0.28	4.45	<0.001	0.15
7a) TAS (F1)	0.29	0.21	0.37	6.76	<0.001	0.22
7b) TAS (F2)	0.15	0.07	0.24	3.47	<0.001	0.11
7c) TAS (F3)	–0.01	–0.10	0.08	–0.23	0.817	0.01
8) BDI	0.19	0.11	0.28	4.43	<0.001	0.15
8a) BDI (F1)	0.20	0.12	0.29	4.66	<0.001	0.15
8b) BDI (F2)	0.15	0.07	0.24	3.47	<0.001	0.11
9) MAAS	–0.02	–0.11	0.07	–0.46	0.644	0.02
10) MSPQ	0.20	–0.12	0.28	4.60	<0.001	0.15

*BICI, Body Image Concern Inventory; BICI (F1), Factor 1 “dysmorphic symptoms”; BICI (F2), Factor 2 “symptom interference”; RSES, Rosenberg Self-Esteem Scale; GSE, General Self-Esteem; BAQ, Body Awareness Questionnaire; WHYMPI-S, West Haven–Yale Multidimensional Pain Inventory – Short Version; TAS20, 20-item Toronto Alexithymia Scale; TAS20 (F1), Factor 1 “difficulty identifying feelings and distinguishing between feelings and bodily sensations in emotional activation”; TAS20 (F2), Factor 2 “difficulty in the verbal expression of emotions”; TAS20 (F3): Factor 3 “externally oriented thinking”; BDI-II: Beck Depression Inventory II; BDI-II (F1): factor 1 “cognitive–affective subscale”; BDI-II (F2): factor 2 “somatic–performance subscale”; MAAS, Mindfulness Attention Awareness Scale; MSPQ, Modified Somatic Perception Questionnaire.*

### General Linear Model

The results of the MANOVA revealed no significant differences regarding gender or age on level of postural awareness (Tables 7, 8).

**TABLE 7:** Summary of PAS total scale and PAS subscales scores by men and women.

				95% Confidence interval		
Dependent variable	Sex	Mean	Standard error	Lower	Upper	Partial η ^2^
PAS	Male	44.97^a^	0.97	43.06	46.87	0.000
	Female	44.35^a^	1.00	42.39	46.31	
PAS (F1)	Male	22.99^a^	0.66	21.70	24.29	0.000
	Female	22.98^a^	0.68	21.65	24.31	
PAS (F2)	Male	21.98^a^	0.67	20.65	23.30	0.000
	Female	21.36^a^	0.69	20.01	22.72	

*^*a*^Based on modified population marginal mean. PAS, Postural Awareness Scale; PAS (F1), Factor 1 “need for attention regulation with postural awareness”; PAS (F2), Factor 2 “ease/familiarity with postural awareness.”*

**TABLE 8:** Summary of PAS total scale and PAS subscales scores in different age range.

				95% Confidence interval		
Dependent variable	Age range	Mean	Standard error	Lower	Upper	Partial η^2^
PAS	18–24	42.942^a^	1.099	40.786	45.099	0.008
	25–34	42.873^a^	1.220	40.478	45.268	
	35–44	44.350^a^	1.637	41.138	47.563	
	45–54	45.327^a^	1.885	41.626	49.027	
	> 54	48.658^a^	2.090	44.555	52.761	
PAS (F1)	18–24	22.354^a^	0.745	20.891	23.817	0.006
	25–34	23.279^a^	0.828	21.654	24.904	
	35–44	23.385^a^	1.111	21.205	25.564	
	45–54	21.597^a^	1.279	19.085	24.108	
	> 54	24.383^a^	1.418	21.599	27.167	
PAS (F2)	18–24	20.588^a^	0.761	19.095	22.081	0.010
	25–34	19.594^a^	0.845	17.935	21.252	
	35–44	20.966^a^	1.133	18.741	23.190	
	45–54	23.730^a^	1.306	21.167	26.293	
	> 54	24.275^a^	1.448	21.434	27.117	

*^*a*^Based on modified population marginal mean. PAS, Postural Awareness Scale; PAS (F1), Factor 1 “need for attention regulation with postural awareness”; PAS (F2), Factor 2 “ease/familiarity with postural awareness.”*

There was a significant difference between those who practice sport and those who do not (Table 9) when considered jointly on the variables total PAS, PAS (F1) and PAS (F2), Wilk’s Λ = 0.991, *F*(2,843) = 3.93, *p* = 0.020, partial η^2^ = 0.01. A separate ANOVA was conducted for each dependent variable, with each ANOVA evaluated at an α level of 0.025. There were significantly higher scores in those who practice sport than those who do not on both total PAS score and first PAS subscale, but not on the second one: *F*(1,844) = 7.80, *p* = 0.005, partial η^2^ = 0.01; *F*(1,844) = 5.87, *p* = 0.028, partial η^2^ = 0.01, respectively.

**TABLE 9:** Summary of PAS total scale and PAS subscales scores by people who practice or not sport.

				95% Confidence interval		
Dependent variable	Sport activity	Mean	Standard error	Lower	Upper	Partial η ^2^
PAS	No	42.725^a^	1.003	40.757	44.693	0.009
	Yes	46.557^a^	0.973	44.648	48.466	
PAS (F1)	No	21.987^a^	0.680	20.652	23.322	0.006
	Yes	23.986^a^	0.660	22.691	25.282	
PAS (F2)	No	20.738^a^	0.694	19.376	22.101	0.004
	Yes	22.571^a^	0.673	21.249	23.893	

*^*a*^Based on modified population marginal mean. PAS, Postural Awareness Scale; PAS (F1), Factor 1 “need for attention regulation with postural awareness”; PAS (F2), Factor 2 “ease/familiarity with postural awareness.”*

Indeed, significant differences related to BMI (Table 10) were found when considered jointly on the variables total PAS, PAS (F1) and PAS (F2), Wilk’s Λ = 0.980, *F*(8,1686) = 1.12, *p* = 0.031, partial η^2^ = 0.01. A separate ANOVA was conducted for each dependent variable, with each ANOVA evaluated at an α level of 0.025. There was a significant difference among the different BMI range only on total PAS score: *F*(4,844) = 2.38, *p* = 0.050, partial η^2^ = 0.01. More specifically, *post hoc* analysis (Scheffé) showed that the group “normal weight” had a higher level of postural awareness.

**TABLE 10:** Summary of PAS total scale and PAS subscales scores in different BMI range.

				95% Confidence interval			
Dependent variable	BMI range	Mean	Standard Error	Lower	Upper	Partial η^2^	Scheffé *post hoc*
PAS	Underweight	45.757^a^	2.085	41.664	49.849	0.011	G2 > G1 >
	Normal weight	47.482	0.877	45.762	49.203		G3 > G4 >
	Overweight	44.603	1.047	42.548	46.657		G5
	Class I obesity	43.058^a^	1.589	39.938	46.177		
	Classes II and III obesity	41.718^a^	2.527	36.758	46.678		
PAS (F1)	Underweight	24.237^a^	1.415	21.461	27.014	0.010	–
	Normal weight	24.010	0.595	22.842	25.177		
	Overweight	21.690	0.710	20.296	23.084		
	Class I obesity	21.671^a^	1.079	19.554	23.788		
	Classes II and III obesity	23.996^a^	1.715	20.631	27.362		
PAS (F2)	Underweight	21.519^a^	1.444	18.685	24.353	0.010	–
	Normal weight	23.472	0.607	22.281	24.664		
	Overweight	22.913	0.725	21.490	24.335		
	Class I obesity	21.386^a^	1.101	19.226	23.547		
	Classes II and III obesity	17.721^a^	1.750	14.287	21.156		

*^*a*^Based on modified population marginal mean. PAS, Postural Awareness Scale; PAS (F1), Factor 1 “need for attention regulation with postural awareness”; PAS (F2), Factor 2 “ease/familiarity with postural awareness”; G1, underweight; G2, normal weight; G3, overweight; G4, Class I obesity; G5, Classes II and III obesity.*

## Discussion

The aim of this research was to analyze the psychometric characteristics of the Italian version of the PAS (Cramer et al., 2018b), a measure of body posture awareness. This tool fits into a perspective that connects posture to well-being (Lauche et al., 2017) and which, in turn, falls within a broader theoretical frame including a growing literature supporting the close link between physical and mental aspects (e.g., Ogden et al., 2012; Van Der Kolk, 2014; Fisher, 2017).

The Italian version of the PAS showed satisfactory psychometric properties with good indications of internal consistency and construct validity. The results obtained with MAP, HPA, and EFA supported a two-factor solution, as confirmed by the CFA and in line with the original version: the first regards the ability to have a high postural awareness in a natural and effortless way (Factor 1 “ease/familiarity with postural awareness”); the second refers the need of high efforts to be aware of their own posture (Factor 2 “need for attention regulation with postural awareness”). In line with what the authors of the original instrument indicated, the two subscales (both with good internal consistency) would seem to indicate the extremes of a continuum concerning the effort employed to be aware of one’s posture (Cramer et al., 2018b).

Positive and significant correlations were found with the mindfulness (MAAS) and the body awareness (BAQ) measurements, although in the relationship with the latter the second factor of the PAS (need for attention regulation with postural awareness) is an exception (in line with the results of the original version, in which there was a low association). The absence of association of this subscale could be interpreted looking at the need of efforts to be aware of his own posture as a difficulty and a lower spontaneity to have mental representation of body aspects. More specifically, the Multiple Code Theory (Bucci, 1999) considers the visceral and physical sensations as subsymbolic processes that, through a referential process, can be depicted within the symbolic register provided by language and images. A lack of integration of these elements does not allow having a full bodily processes awareness, which is a fundamental element for the distinction between emotive or physiological physical activations.

This condition causes tensions and dysregulated states of emotional arousal that could lead to psychosomatic problems (Ruesch, 1948; MacLean, 1949): all this could result in greater attention to somatic aspects, which, however, do not lead to awareness, but only to excessive worry and anxiety. The above is confirmed by the negative associations of postural awareness with alexithymia (especially externally oriented thinking) and the perception of physiological functions linked to states of anxiety and malaise (respectively, TAS-20 and MSPQ, which are instead positively correlated to each other). A lack of integration between symbolic and subsymbolic processes, therefore, does not allow to understand, express, and elaborate the somatic activations. In fact, the data show that both natural focus and active attention aimed at achieving and maintaining high levels of postural awareness are linked with a decrease of negative effect perception from the pain experiences (WHYMPI-S), which is in line with the scientific literature that shows that a higher non-judgmental bodily consciousness is associated with lower physical pain (Zeidan and Vago, 2016; Anheyer et al., 2017) and with a decrease in the anxiety that this condition determines (Flink et al., 2009). Furthermore, regarding the attention to aesthetic features, a negative and worried attitude toward one’s appearance would seem to be associated with a sense of detachment from the body and a complete unwillingness to make efforts to be aware of the posture assumed. Indeed, negative correlations were found between PAS and dysmorphic concern scores (BICI), except for the first factor (in line with the original study).

Positive correlations with self-esteem (RSES) and self-efficacy (GSE) and negative associations with depression (BDI-II) are also identified. Scientific literature supports evidence that certain bodily attitudes can influence self-confidence, the perception of being able to cope with difficulties, and emotional state (Keltner et al., 2003; Michalak et al., 2014; Nair et al., 2015; Cuddy, 2016); on the one hand, it could deduce that a greater posture awareness allows greater control over it and over the states it influences, favoring a more positive self-image; on the other hand, this could also be interpreted taking into account that higher levels of self-esteem and self-efficacy are associated to higher insight (Gori et al., 2015), also allowing a greater sense of mastery in one’s environment and a greater awareness of how body fits and interacts with it, facilitating a state of well-being.

Besides, to have more accurate interpretations about the differences between PAS subscales correlations, an inferential test was used to determine whether relevant pairs of correlations were statistically different in magnitude. The findings support the construct validity: significant associations were found between the positive correlations that the PAS subscales have with the PAS total score, between those with the MAAS and between the negative correlations that they have with the TAS20 “external oriented thinking” subscale.

Other important results obtained from the present research confirm the positive effects of physical activity and healthy body weight. Indeed, previous studies suggest that repeated exposure to bodily functions related to physical activity (e.g., increased breathing and heart rate) may lead to better body awareness in the various aspects that characterize it (Skrinar et al., 1986; Mehling et al., 2009), which in turn can be associated with greater body satisfaction and a decrease in disordered eating attitudes (Daubenmier, 2005). On the contrary, no significant differences were found regarding gender and age, in line with other research (Price and Thompson, 2007; Cramer et al., 2018b). This study has some limitations that need to be identified and discussed. First, several statistical comparisons have been carried out without any control procedure for false discovery rate, and this should be considered in the interpretation of the results: future research could overcome this limit, also correcting the *p*-values for multiple comparisons. Besides, as self-report tool, the PAS requires a self-assessment of aspects for which there could be a low level of consciousness; by definition, it is not possible to understand the actual association between self-report and the real postural awareness. Future research could use a multimodal approach (e.g., adding laboratory measurements and in-depth interviews) to have more complete assessments and overcome this limit, albeit with a greater expenditure of resources. Furthermore, the sample is composed only of Italian subjects, and this impacts the generalizability of the results in other cultures. Specifically, it could be interesting to study and analyze the differences in postural awareness levels in Eastern countries, considering, for example, the positive impact that different martial arts having their origin and diffusion have on this aspect (e.g., Lauche et al., 2017). Thus, future research could expand the sample by including employees from different geographical areas, to test the cross-cultural invariance of the results too.

Despite these limitations, the results of this validation study suggest that the Italian version of the PAS is a rapid tool, simple in its administration and evaluation, and with good psychometric properties; these data imply the possibility of using this self-report easily both for research and clinical practice, elaborating interventions within the psychotherapeutic process that can act on the two dimensions of the postural awareness construct (“need for attention regulation with postural awareness” and “ease/familiarity with postural awareness”).

## Data Availability Statement

The raw data supporting the conclusions of this article will be made available by the authors, without undue reservation, to any qualified researcher.

## Ethics Statement

Ethical review and approval was not required for the study on human participants in accordance with the local legislation and institutional requirements. The patients/participants provided their written informed consent to participate in this study.

## Author Contributions

All authors conceptualized and designed the study, collaborated for the realization of the back-translation, contributed to manuscript revision, read, and approved the submitted version. ET recruited the subjects, administered them the protocol, and wrote the first draft of the manuscript. AG and ET performed the data analysis. AG was the scientific supervisor of the study.

## Conflict of Interest

The authors declare that the research was conducted in the absence of any commercial or financial relationships that could be construed as a potential conflict of interest.
